# Combined Metabolic Activators with Different NAD+ Precursors Improve Metabolic Functions in the Animal Models of Neurodegenerative Diseases

**DOI:** 10.3390/biomedicines12040927

**Published:** 2024-04-22

**Authors:** Ozlem Altay, Hong Yang, Serkan Yildirim, Cemil Bayram, Ismail Bolat, Sena Oner, Ozlem Ozdemir Tozlu, Mehmet Enes Arslan, Ahmet Hacimuftuoglu, Saeed Shoaie, Cheng Zhang, Jan Borén, Mathias Uhlén, Hasan Turkez, Adil Mardinoglu

**Affiliations:** 1 Science for Life Laboratory, KTH—Royal Institute of Technology, 171 65 Stockholm, Sweden; oaltay@kth.se (O.A.); hong.yang@scilifelab.se (H.Y.); cheng.zhang@scilifelab.se (C.Z.); mathias.uhlen@scilifelab.se (M.U.); 2 Department of Pathology, Faculty of Veterinary Medicine, Atatürk University, Erzurum 25240, Turkey; syildirim@atauni.edu.tr (S.Y.); ismail.bolat@atauni.edu.tr (I.B.); 3 Department of Pharmacology and Toxicology, Faculty of Veterinary Medicine, Atatürk University, Erzurum 25240, Turkey; cemil489@gmail.com; 4 Department of Molecular Biology and Genetics, Faculty of Science, Erzurum Technical University, Erzurum 25240, Turkey; senaoner02@gmail.com (S.O.); ozlem.ozdemir@erzurum.edu.tr (O.O.T.); enesiyte@gmail.com (M.E.A.); 5 Department of Medical Pharmacology, Faculty of Medicine, Atatürk University, Erzurum 25240, Turkey; ahmeth@atauni.edu.tr; 6 Centre for Host-Microbiome Interactions, Faculty of Dentistry, Oral & Craniofacial Sciences, King’s College London, London SE1 9RT, UK; saeed.shoaie@kcl.ac.uk; 7 Department of Molecular and Clinical Medicine, Sahlgrenska University Hospital, University of Gothenburg, 413 45 Gothenburg, Sweden; jan.boren@wlab.gu.se; 8 Department of Medical Biology, Faculty of Medicine, Atatürk University, Erzurum 25240, Turkey; hasanturkez@yahoo.com

**Keywords:** Alzheimer’s disease, Parkinson’s disease, combined metabolic activators, animal models

## Abstract

Background: Mitochondrial dysfunction and metabolic abnormalities are acknowledged as significant factors in the onset of neurodegenerative disorders such as Parkinson’s disease (PD) and Alzheimer’s disease (AD). Our research has demonstrated that the use of combined metabolic activators (CMA) may alleviate metabolic dysfunctions and stimulate mitochondrial metabolism. Therefore, the use of CMA could potentially be an effective therapeutic strategy to slow down or halt the progression of PD and AD. CMAs include substances such as the glutathione precursors (L-serine and N-acetyl cysteine), the NAD+ precursor (nicotinamide riboside), and L-carnitine tartrate. Methods: Here, we tested the effect of two different formulations, including CMA1 (nicotinamide riboside, L-serine, N-acetyl cysteine, L-carnitine tartrate), and CMA2 (nicotinamide, L-serine, N-acetyl cysteine, L-carnitine tartrate), as well as their individual components, on the animal models of AD and PD. We assessed the brain and liver tissues for pathological changes and immunohistochemical markers. Additionally, in the case of PD, we performed behavioral tests and measured responses to apomorphine-induced rotations. Findings: Histological analysis showed that the administration of both CMA1 and CMA2 formulations led to improvements in hyperemia, degeneration, and necrosis in neurons for both AD and PD models. Moreover, the administration of CMA2 showed a superior effect compared to CMA1. This was further corroborated by immunohistochemical data, which indicated a reduction in immunoreactivity in the neurons. Additionally, notable metabolic enhancements in liver tissues were observed using both formulations. In PD rat models, the administration of both formulations positively influenced the behavioral functions of the animals. Interpretation: Our findings suggest that the administration of both CMA1 and CMA2 markedly enhanced metabolic and behavioral outcomes, aligning with neuro-histological observations. These findings underscore the promise of CMA2 administration as an effective therapeutic strategy for enhancing metabolic parameters and cognitive function in AD and PD patients.

## 1. Introduction

Neurodegenerative diseases (NDDs), including Parkinson’s disease (PD) and Alzheimer’s disease (AD), are increasingly recognized for their complex interplay with metabolic abnormalities [1]. These disorders not only impair motor and cognitive functions but also involve significant alterations in metabolic pathways critical for brain health. For instance, mitochondrial dysfunction contributes to neuronal death and the progression of both disorders [2]. It also influences other critical cellular processes such as calcium homeostasis and apoptotic pathways, further exacerbating neuronal damage [3,4]. 

In recent years, a multitude of studies have revealed links between metabolic abnormalities and the mechanisms of disease within the brain [3,5,6,7,8,9,10,11]. These alterations affect crucial mitochondrial components leading to impaired cellular energy production and increased oxidative stress, thereby contributing to the neurodegenerative process [12,13,14,15,16,17,18,19]. The relevance of neuroprotective strategies in managing NDDs is increasingly recognized, particularly through mechanisms that reduce inflammatory processes. Such strategies are crucial in slowing the progression of diseases such as AD and other NDDs. Among the promising approaches is the use of precursors to nicotinamide adenine dinucleotide (NAD+) [10]. Supplementation with NAD+ is shown to be beneficial in improving metabolic impairments that are closely linked to aging and the onset of associated diseases [3,11]. This is particularly pertinent, as mitochondrial impairment has been identified as a biological basis for the decreased brain function observed in NDDs. Therefore, targeting mitochondrial dysfunction presents a novel and potentially effective avenue for the treatment of neurodegenerative conditions, emphasizing the importance of neuroprotective strategies in mitigating the impact of these debilitating diseases.

The utilization of animal models to explore the intricacies of NDDs has illuminated the detrimental impact of a high-fat diet (HFD) on cognitive regions of the brain that are pivotal in NDDs, manifesting as memory impairment and neuroinflammation [20,21,22,23]. Furthermore, the association of HFD with increased permeability of the blood–brain barrier, along with motor deficits and behavioral changes in rodent models, underscores the potential of dietary factors in influencing NDD pathogenesis [24]. These observations emphasize the value of animal models that closely mimic human physiological responses, as they provide a critical window into the biological underpinnings of NDDs precipitated by dietary choices [25]. 

The intricacies of diseases affecting brain metabolism demand a multifaceted therapeutic approach, as targeting a single metabolic pathway proves insufficient [26]. This complexity is mirrored in the liver’s metabolic challenges, particularly the insufficiency of glutathione (GSH) levels critical for maintaining the thiol redox status [27,28]. Restoration of GSH levels through L-serine and N-acetylcysteine administration underscores the potential of precise metabolic interventions [27,28]. This approach is further refined in the development of combined metabolic activators (CMA1), comprising L-serine, N-acetyl cysteine (NAC), nicotinamide riboside (NR), and L-carnitine tartrate (LCAT). These compounds, selected through integrative network analysis of non-alcoholic fatty liver disease (NAFLD), are instrumental in stimulating mitochondria, enhancing fatty acid oxidation, and mitigating inflammation, demonstrating efficacy in both animal models and human subjects [27,29,30,31,32,33] A particular study utilizing liver transcriptomics from a NAFLD hamster model, analyzed through a genome-scale metabolic model, elucidates the CMA’s ability to activate mitochondria and modulate metabolic pathways [34]. 

In a recent study, we evaluated the therapeutic potential of metabolic activators in the treatment of AD and PD animal models [35]. This investigation is premised on the hypothesis that metabolic activation can enhance mitochondrial function and thereby improve the metabolic health of patients with these conditions. We found considerable damage in the brain and liver tissues of those on a high-fat diet compared to those on a standard chow diet. Histological analysis showed that the administration of CMA (same composition as CMA1) led to improvements in hyperemia, degeneration, and necrosis in neurons for both AD and PD models. This was further corroborated by immunohistochemical data, which indicated a reduction in immunoreactivity in the neurons. Additionally, notable metabolic enhancements in liver tissues were observed. In PD rat models, CMA treatment also appeared to positively influence behavioral functions [35].

Recently, we evaluated the acute metabolic responses to metabolic activators supplemented with either cysteine or NAC, in conjunction with or without nicotinamide or niacin, through a longitudinal metabolomics analysis [36]. Seventy healthy volunteers were administered CMA in a placebo-controlled design to assess the impact on plasma metabolomics profiles. The investigation revealed that CMAs, regardless of the specific metabolic activators used, induced significant alterations in metabolic pathways related to amino acid, lipid, and nicotinamide metabolism. Notably, CMA supplementation with cysteine demonstrated a well-tolerated and safe profile, comparable to that observed with NAC supplementation. Furthermore, the inclusion of nicotinamide as a component of CMA formulations highlighted its potential to enhance the metabolic response, particularly in pathways relevant to NAD+ biosynthesis [37]. These findings underscore the therapeutic potential of carefully selected CMA formulations in modulating metabolic pathways, offering insights into their role in managing and potentially treating metabolic disorders. Moreover, we evaluated the safety and acute effects of six different CMAs combined with 1 g of various NAD+ precursors through global metabolomics analysis in a one-day double-blind, placebo-controlled human clinical trial. We found that the NAD+ salvage pathway primarily enhances NAD+ levels upon administering CMAs without NAD+ precursors. We observed that adding nicotinamide (NAM) to CMAs most effectively increased NAD+ levels, followed by niacin, NR, and nicotinamide mononucleotide (NMN), whereas flush-free niacin (FFN) did not have this effect. The study concluded that CMAs containing NAM, NMN, and NR are effective in boosting NAD+ levels to improve metabolic conditions, providing a comprehensive plasma metabolomic profile of different CMA formulations. 

Here, we evaluated the effect of CMA1 (nicotinamide riboside, L-serine, N-acetyl cysteine, L-carnitine tartrate) and CMA2 (nicotinamide, L-serine, N-acetyl cysteine, L-carnitine tartrate) as well as their individual components on the animal models of AD and PD. The results from CMA1 and its individual components have been reported earlier [35]. In this study, we conducted a comprehensive analysis of the effects of CMA1 and CMA2 administration on various parameters in the animal models of NDDs, focusing on metabolic health, behavior, and histological changes in liver and brain tissues.

## 2. Materials and Methods

### 2.1. Animal Study Design for AD

The methods are described in our previous study [35]. Briefly, female Sprague-Dawley rats, aged 12 weeks and weighing between 320 and 380 g, were housed in a controlled environment with a temperature of 22 ± 2 °C and humidity set at 50 ± 5%. They experienced a 12 h light/dark cycle within their cages, with unrestricted access to food and water. Rats exhibiting symptoms such as reduced appetite or pain, which would violate animal ethics guidelines as determined by a veterinarian, were excluded from the study. This research was carried out at the Medical Experimental Research Center of Atatürk University in Erzurum, Turkey. To maintain objectivity, investigators, pathologists, and a statistician were kept uninformed about the study’s specifics. The Ethical Committee of Atatürk University sanctioned all animal treatment procedures (approval from the Atatürk University Experimental Research Center, Erzurum, Turkey, dated 31.05.2022/No:103), ensuring adherence to the National Institutes of Health’s standards for the Care and Use of Laboratory Animals.

**Figure 1:**
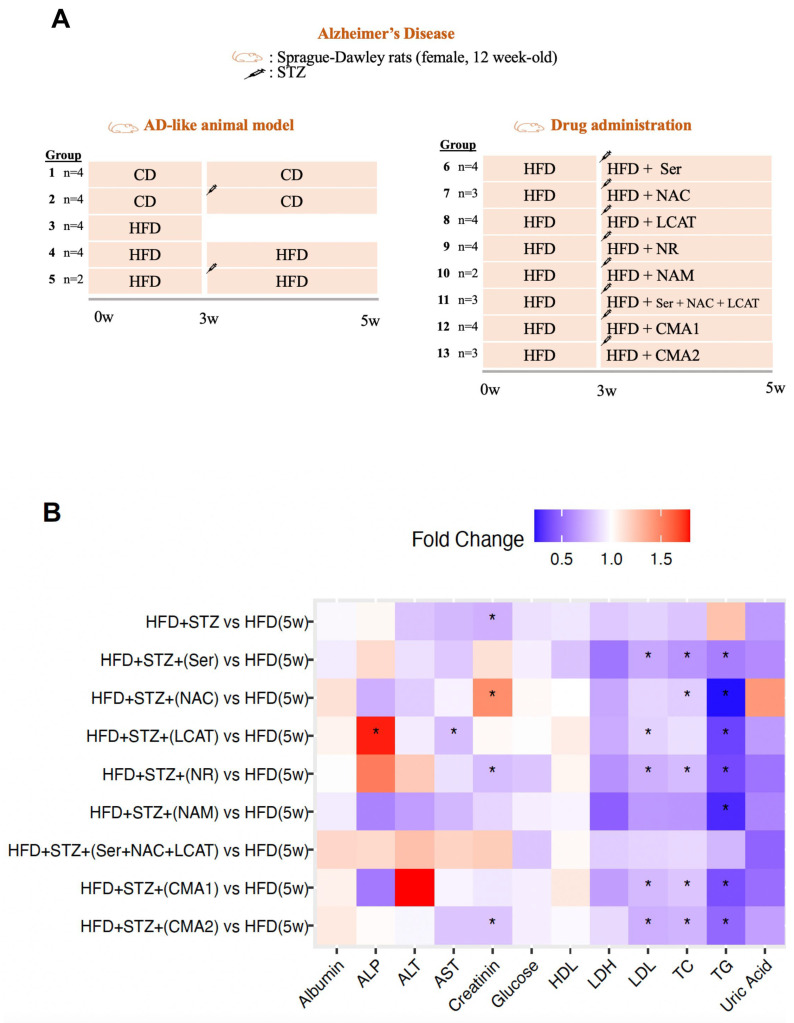
The effect of CMA administration in AD-like animal model. (**A**) Overview of the study design and group information. The left side of the image describes how the AD-like animal model was generated. Group 1 (n = 4) was fed with chow diet (CD) for 5 weeks; Group 2 (n = 4) was fed with CD and treated with streptozotocin (STZ). The syringe icon shows the STZ injection in the third week. Group 3 (n = 4) was fed with a high-fat diet (HFD) for only 3 weeks, and group 4 (n = 4) was fed with HFD for 5 weeks. Group 5 (n = 2) was fed with HFD for 5 weeks and treated with streptozotocin (STZ). The right side of the image describes a drug administration study. Groups 6–13 were fed with HFD for 5 weeks. In the third week, animals were treated with STZ and administered with individual or combined metabolic activators for 2 weeks. (**B**) Heatmap shows FC-based relative alterations of the clinical variables in the rat study groups. Asterisks (*) indicate statistical significance based on one-way ANOVA or Mann–Whitney U test, *p*-value < 0.05 is considered statistical significance. HFD: high-fat diet, Ser: L-serine, NAC: N-acetyl-L-cysteine, LCAT: L-carnitine tartrate, NR: nicotinamide riboside, NAM: nicotinamide, TG: triglyceride, TC: total cholesterol, ALP: alkaline phosphatase, AST: aspartate aminotransferase, ALT: alanine aminotransferase, HDL: high-density lipoprotein, LDL: low-density lipoprotein, LDH: lactate dehydrogenase, CMA1: Combined Metabolic Activators 1; CMA2: Combined Metabolic Activators 2; FC: Fold change. The results from CMA1 and its individual components have been reported earlier [35].

After one week of habituation to lab settings, a set of rats was allocated into two distinct groups following a completely randomized method. The AD model in rats is generated by STZ (Sigma-Aldrich, Darmstadt, Germany) induction. After sedating the rats with a ketamine/xylazine mixture (60/10 mg/kg b.w.), they were secured in a stereotaxic apparatus (Stoelting, IL, USA). Using precise coordinates from Paxinos and Watson’s guidelines, the STZ was injected into the appropriate location in the brain. The exact coordinates used for the injection were 0.8 mm posterior to bregma, 1.5 mm bilateral to the sagittal suture, and 3.6 mm below the surface of the skull. The STZ solution (3 mg/kg b.w.) was administered for 4 min. To ensure that the solution did not leak out, the needle was left in place for an additional 4 min after the injection. The dietary intake consisted of either a standard chow diet (CD, for Groups 1–2) or HFD (Groups 4–13) for five weeks. Specifically, Group 3 was provisioned with an HFD for three weeks and then culled to ascertain the model’s establishment. The investigation encompassed a total of 45 rats. The first group served as the baseline for the HFD, whereas the fourth group was used as the control for pharmacological investigations. Following three weeks, animals in Groups 6–13 were subjected to STZ treatment and received either individual or combined metabolic activators over two weeks via oral gavage. Group 5 underwent treatment solely with STZ; Group 6 was administered with L-serine at a dose of 1000 mg/kg daily (Ajinomoto Health & Nutrition North America, Inc., Raleigh, NC, USA); Group 7 with NAC at 300 mg/kg daily (Wuhan Grand Hoyo Co., Ltd., Hubei, China); Group 8 with LCAT at 100 mg/kg daily (Health Biochem Technology Co., Ltd., Xi’an, China); Group 9 with NR at 120 mg/kg daily (ChromaDex Inc., Los Angeles, CA, USA); Group 10 with NAM at 120 mg/kg daily (Health Zhongrui Pharmaceutical Co., Ltd., Tianjin, China); Group 11 with a combination of L-serine, NAC, and LCAT; Group 12 with CMA1, a combination of L-serine, NAC, LCAT, and NR; and Group 13 with CMA2, a combination of L-serine, NAC, LCAT, and NAM (Figure 1A). All treatment is given through oral gavage. Rat weights were logged weekly (Dataset S1). Following the 5-week period, the rats were anesthetized using isoflurane and euthanized. Blood was then drawn from the abdominal aorta, followed by centrifugation at 8000 rotations per minute for a quarter hour at a temperature of 4 °C. This process was to conduct biochemical analyses of the blood, which were performed with an automated chemical analysis machine. Organs such as the heart, fat stores, liver, kidneys, brain, muscles, and segments of the intestine, as well as the pancreas, colon, and stomach, were promptly excised, instantly preserved in a liquid nitrogen environment, and subsequently kept at −80 °C. 

### 2.2. Animal Study Design for PD

*Wistar* rats aged 12 weeks and weighing 320–380 g were housed at a consistent 22 ± 2 °C with 55% humidity and a 12 h light-dark cycle. They had free access to food and water. The handling of animals was approved by the Atatürk University’s Ethics Committee (approval from Atatürk University’s Experimental Research Center, Erzurum, Turkey, on 31 May 2022, Reference Number: 103) and followed the National Institutes of Health’s animal care guidelines. The study’s details were undisclosed to researchers, pathologists, and statisticians to maintain impartiality. Animals that exhibited reduced appetite, pain, or other distressing symptoms as assessed by veterinarians were excluded. Chemicals such as 6-hydroxydopamine hydrochloride (6-OHDA), L-ascorbic acid, and apomorphine hydrochloride were procured from Sigma-Aldrich Co. LLC in Steinheim am Albuch, Germany. A high-fat diet for the rats was provided by HEKA Science, Istanbul, Turkey.

Following a week of adjustment to the lab environment, the rats were split into groups using a completely randomized design to assess the effect of CMA1 and CMA2 and their components on brain and liver tissues in a PD-like model, induced by 6-OHDA. They were evaluated based on pathological changes and behavior tests. Group 1 was given a CD for four weeks. Group 2 received CD along with 6-OHDA treatment. Group 3 rats were fed an HFD for the same duration, while Group 4 had HFD coupled with 6-OHDA. The study included 46 rats in total. Group 1 served as the control for the HFD, and Group 4 was the control for drug studies. Rats in Groups 5–12 were given HFD and 6-OHDA treatment. Two weeks into the study, these groups were also given CMA and individual components for an additional two weeks. Groups 5–9 were treated with one of the components at a specified dosage daily (L-serine, 1000 mg/kg; NAC, 300 mg/kg; LCAT, 100 mg/kg; NR, 120 mg/kg; NAM, 120 mg/kg; respectively). Group 10 was given a combination of L-serine, NAC, and LCAT, Group 11 received CMA1, which included L-serine, NAC, LCAT, and NR. Lastly, Group 12 was treated with CMA2, which is a mix of L-serine, NAC, LCAT, and NAM. All treatments were administered orally once daily.

The process to induce a PD-like state in rats involved the administration of 6-OHDA following protocols established in previous studies. After sedating the rats with a ketamine/xylazine mixture (60/10 mg/kg body weight), they were secured in a stereotaxic apparatus (Stoelting, IL, USA). Using precise coordinates from Paxinos and Watson’s guidelines, the 6-OHDA was injected into the appropriate location in the brain. The exact coordinates used for the injection were 5.5 mm posterior to bregma, 2 mm lateral to the sagittal suture, and 8 mm below the surface of the skull. The 6-OHDA solution (8 μg/μL at a concentration of 4 μL in saline mixed with 0.01% ascorbic acid to prevent oxidation) was administered for 4 min. To selectively target 6-OHDA to dopaminergic neurons, a selective and noradrenergic reuptake inhibitor, desipramine (30 mg/kg), was administered. To increase the striatal dopamine loss, 10 mg/kg pargyline was also administered. Additionally, to protect other neurons in the brain, 6-OHDA was prepared in a 0.1% ascorbic acid solution and stored in a dark tube before performing injections. To ensure that the solution did not leak out, the needle was left in place for an additional 4 min after the injection.

#### 2.2.1. Histopathological, Immunohistochemical and Immunofluorescence Examination

Liver and brain tissue samples from the experimental study were processed for histopathological examination. The samples were first fixed in a 10% buffered formalin solution for 48 h. They were then embedded in paraffin, and 4 µm sections were cut from each paraffin block. For histological analysis, the sections were stained with hematoxylin and eosin (HE) and observed under an Olympus BX51 light microscope (Olympus, Germany). Two independent pathologists assessed the tissue sections according to a predefined histopathological scale ranging from none (−) to very mild (+), mild (++), moderate (+++), severe (++++), and very severe (+++++). Further immunoperoxidase studies were conducted on sections placed on poly-L-lysin-coated slides. After deparaffinization and dehydration, the tissues underwent endogenous peroxidase suppression with 3% H_2_O_2_ and antigen retrieval by boiling in a retrieval solution. To minimize nonspecific background staining, a protein block was applied to the sections for 5 min before the addition of primary antibodies. For brain tissue samples, an antibody targeting 8-OHdG (Cat No: sc-66036, Santa Cruz, CA, USA; dilution 1/100) was used, while for liver tissues, the primary antibodies were JNK1/3 and Caspase 3 (Cat no: sc-514539; sc-56053, Santa Cruz, CA, USA; dilution 1/100). The 3-3′-diaminobenzidine (DAB) chromogen served as the chromogen for the reaction. After staining, the sections were examined under a fluorescent microscope (ZEISS, Oberkochen, Germany) and analyzed with the ZEISS Zen Imaging Software (ZEN2 Core Axiovision version 1.00), following the manufacturer’s recommendations.

Tissue sections prepared on poly-L-lysin adhesive slides for immunoperoxidase staining went through a sequence of precise preparatory steps. These steps were crucial to preparing the tissues for immunostaining, which would reveal specific antigens using antibodies. Initially, the tissues were deparaffinized and dehydrated. They were then treated with 3% H_2_O_2_ to quench endogenous peroxidase activity, followed by boiling in an antigen retrieval solution. A protein block was applied to prevent non-specific staining and incubated for 5 min. In the case of liver tissues, they were incubated with the primary antibody 8-OHdG (Cat No: sc-66036, Santa Cruz, CA, USA; dilution 1/100) and secondary FITC (Cat No: ab6717, Abcam, Cambridge, UK; Dilution Ratio: 1/500), as well as a separate incubation with H2A.X (Cat No: 10856-1, proteintech, Manchester, UK; dilution ratio: 1/100) primary antibody and Texas Red (Cat No: sc-3917, Santa Cruz, CA, USA; dilution ratio: 1/100) secondary antibody. For brain tissues, NeuN (Cat No: ab104225, Abcam, UK; dilution ratio: 1/100) and Caspase 3 (Cat No: sc-56053, Santa Cruz, CA, USA; Dilution Ratio:1/100) primary antibody and FITC (Cat No: ab6717, Abcam, UK; dilution ratio: 1/500) secondary antibody were used in one step, and Caspase 3 (Cat No: sc-56053, Santa Cruz, CA, USA; dilution ratio: 1/100) and H2A.X (Cat No: 10856-1, proteintech, UK; dilution ratio: 1/100) primary antibody and Texas Red secondary antibody in another. After these incubations, DAPI (Cat No: D-1306, Fisher Scientific, Waltham, MA, USA; dilution ratio: 1/200) was applied to stain the cell nuclei, and the slides were sealed with glycerin. Finally, the stained sections were examined under a fluorescent microscope (ZEISS, Oberkochen, Germany) and analyzed using ZEISS Zen Imaging Software Software (ZEN2 Core Axiovision version 1.00) following the manufacturer’s guidelines. This process allowed for the visualization of specific cellular components and the assessment of pathological changes at a molecular level in the tissues.

#### 2.2.2. Behavioral Test in the PD-like Animal Model

The rats in the study were assessed for motor abilities and drug-induced behavioral responses without the observer knowing which treatments the animals had received. Locomotor activity was tracked using a square plexiglas enclosure with video monitoring. Each rat was placed in the center of this square and monitored for five minutes, during which their movements across the square, pauses, and vertical movements were all logged. Additionally, the response to apomorphine, a drug that induces unusual turning behavior in rats with a PD-like condition, was evaluated. After receiving a dose of apomorphine hydrochloride (2.5 mg/kg intraperitoneally), each rat was observed in a square cage. The researchers counted the number of counter-clockwise rotations, which is a typical response to the medication in the modeled condition, for 30 min.

### 2.3. Statistical Analysis

Data are presented as the mean ± the standard deviation (SD) for continuous variables. The Shapiro–Wilk test was employed to assess the normality of the data distribution. For continuous variables that were normally distributed, one-way ANOVA was utilized, whereas the Mann–Whitney U test was applied for those with a non-normal distribution. A *p*-value of less than 0.05 was deemed to indicate statistical significance. There was no predetermination of the statistical sample size. All statistical analyses were performed using R (version 4.1.3).

## 3. Results

### 3.1. Both CMA1 and CMA2 Improve Metabolic Functions in AD Animal Model

To investigate the impact of CMA1 and CMA2, individual metabolic activators were provided to rats modeled with AD following intracerebroventricular injections of STZ (Figure 1A). It was noted that the combined or individual application of CMA1 and CMA2 components notably reduced (*p* < 0.05) the levels of triglycerides (TG) in the plasma when compared to the baseline group (Group 4; Figure 1B, Dataset S1). Concurrently, the exclusive administration of L-serine or NR led to a substantial decrease in the total cholesterol levels (*p* < 0.05) and in the concentration of low-density lipoprotein (LDL; *p* < 0.05) in these rodents (Figure 1B). Furthermore, notable declines in both total cholesterol (*p* = 0.04) and LDL (*p* = 0.03) were also seen in rats treated with NAC and LCAT, respectively (Figure 1B). Particularly, only in the LCAT-administered rats were significant reductions in plasma AST (*p* = 0.03) and a rise in plasma ALP (*p* = 0.04) levels observed (Figure 1B).

**Figure 2:**
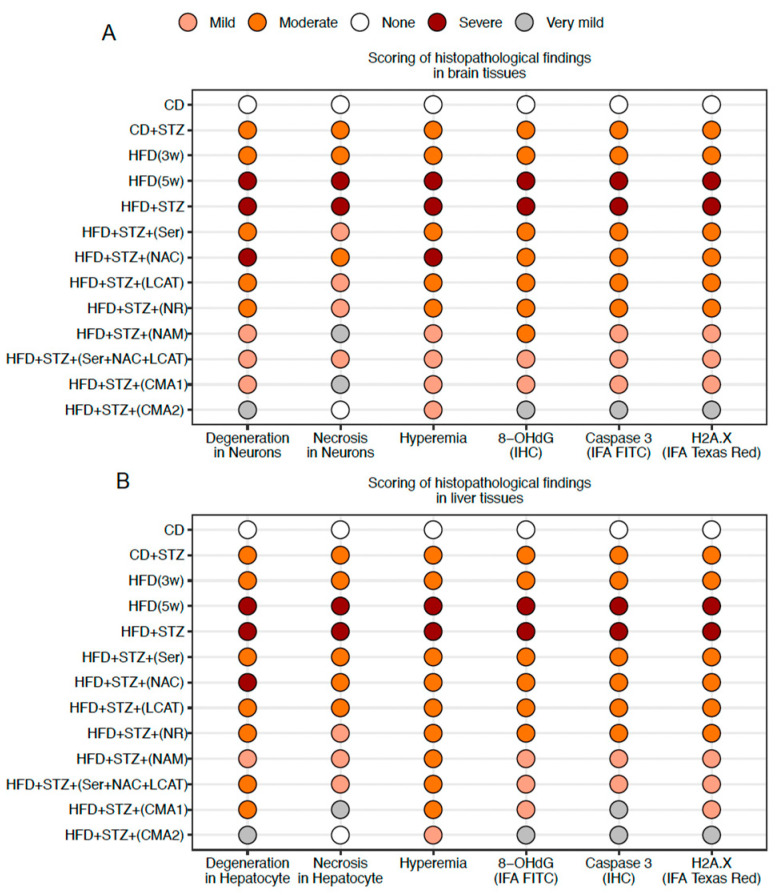
Histopathological, immunohistochemical and immunofluorescence images of rat tissues in the AD-like animal model. Histopathological image analysis results of rat (**A**) brain and (**B**) liver tissue. Slides evaluated by two independent pathologists and immunopositivity scores were: None (−), very mild (+), mild (++), moderate (+++), severe (++++), and very severe (+++++). HFD: high-fat diet, STZ: streptozotocin, Ser: L-serine, NAC: N-acetyl-L-cysteine, LCAT: L-carnitine tartrate, NR: nicotinamide riboside, NAM: nicotinamide, CMA1: Combined Metabolic Activators 1, CMA2: Combined Metabolic Activators 2. The results from CMA1 and its individual components have been reported earlier [35].

The rodent models facilitated the analysis of histopathological variations among the groups and allowed for the evaluation of brain tissue reactions following CMA1 and CMA2 treatment compared to the HFD + STZ group (Figure 2A and Figure S1, Dataset S1). It was observed that the detrimental effects, including hyperemia, degeneration, and necrosis in neurons, were mitigated by the administration of individual compounds as well as their collective combination, CMA. However, a more pronounced recovery was noted with the administration of CMA2 compared to CMA1 and their individual metabolic activators. This improvement was further corroborated by the reduced neuronal immunoreactivity demonstrated through immunohistochemical analysis (Figure 2A and Figure S1, Dataset S1). Remarkably, rats treated with CMA2 showed no neuronal necrosis in brain tissue (Figure 2A and Figure S1, Dataset S1).

Alongside brain improvements, significant liver recovery was also noted post-CMA treatment. Observations indicated that degeneration in hepatocytes, hyperemia, and necrosis were alleviated when treated with either single agents or CMA1. However, an administration of the compound mixture CMA2 yielded a more marked healing process than what was achieved by the metabolic enhancers alone. This enhanced recovery was evidenced by the immunohistochemical analysis (Figure 2B and Figure S1, Dataset S1). Impressively, in the case of rats treated with CMA2, the liver tissue was completely free from any signs of necrosis and only very mild degeneration in hepatocytes (Figure 2B and Figure S1, Dataset S1).

### 3.2. Both CMA1 and CMA2 Improve Metabolic Functions in PD Animal Model

**Figure 3:**
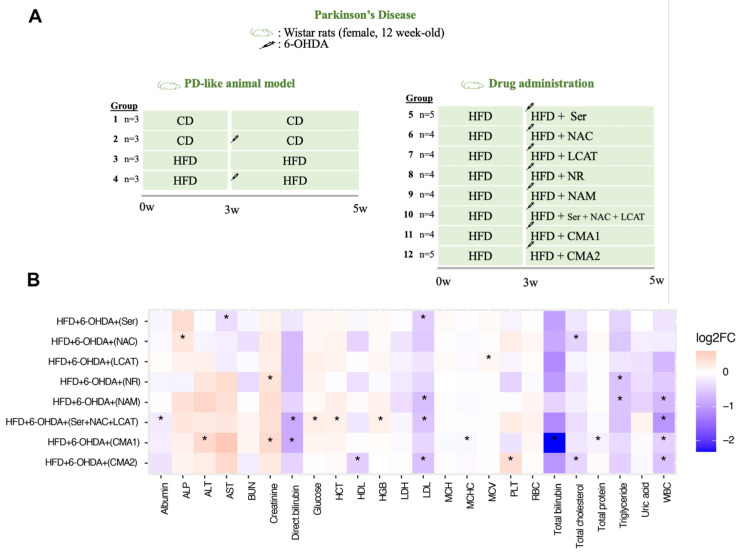
Administration of CMA shows beneficial effects on plasma parameters in PD-like animal models. (**A**) Overview of the study design and group information. The left side of the image describes how the PD-like animal model was generated. Group 1 (n = 3) was fed with a chow diet (CD) for 5 weeks; Group 2 (n = 3) was fed with CD and treated with 6-OHDA. The syringe icon shows the 6-OHDA injection in the third week. Group 3 (n = 3) was fed with a high-fat diet (HFD) for 5 weeks. Group 4 (n = 3) was fed with HFD and treated with 6-OHDA. The right side of the figure describes drug administration studies. Groups 5–12 were fed with HFD for 5 weeks. In the third week, the animals were treated with 6-OHDA and administered with individual or combined metabolic activators for 2 weeks. (**B**) Heatmap plot shows log2FC of biochemical variables between treated groups (administered with individual or CMA) and HFD plus 6-OHDA group. Asterisk (*) denotes statistical significance (*p* < 0.05). The difference and *p*-value are estimated by one-way ANOVA or Mann–Whitney U test. CD: chow diet, 6-OHDA: 6-hydroxydopamine hydrochloride, HFD: high-fat diet, Ser: L-serine, NAC: N-acetyl-L-cysteine, LCAT: L-carnitine tartrate, NR: nicotinamide riboside, NAM: nicotinamide, CMA1: Combined Metabolic Activators 1, CMA2: Combined Metabolic Activators 2, ALP: alkaline phosphatase, ALT: alanine aminotransferase, AST: aspartate transaminase, BUN: blood urea nitrogen, HCT: hematocrit, HDL: high-density lipoprotein, HGB: hemoglobin, LDH: lactate dehydrogenase, LDL: low-density lipoprotein, MCH: mean corpuscular hemoglobin; MCHC: mean corpuscular hemoglobin concentration, MCV: mean corpuscular volume, PLT: platelet (thrombocyte) count, RBC: red blood cell, WBC: white blood cell, log2FC: log transformation fold change. The results from CMA1 and its individual components have been reported earlier [35].

We assessed the effects of metabolic activators in PD rats induced by 6-OHDA injections. (Figure 3A). Our biochemical analysis of metabolic biomarkers revealed notable alterations. Specifically, there was a marked reduction in the levels of bilirubin, AST, LDL, total protein, and white blood cells. Conversely, there was a substantial increase in the ALT, creatinine, glucose, hemoglobin, and hematocrit levels when comparing the treatment groups (those receiving either individual substances or CMA) to the control group, which was given HFD along with 6-OHDA (Figure 3B, Dataset S2).

**Figure 4:**
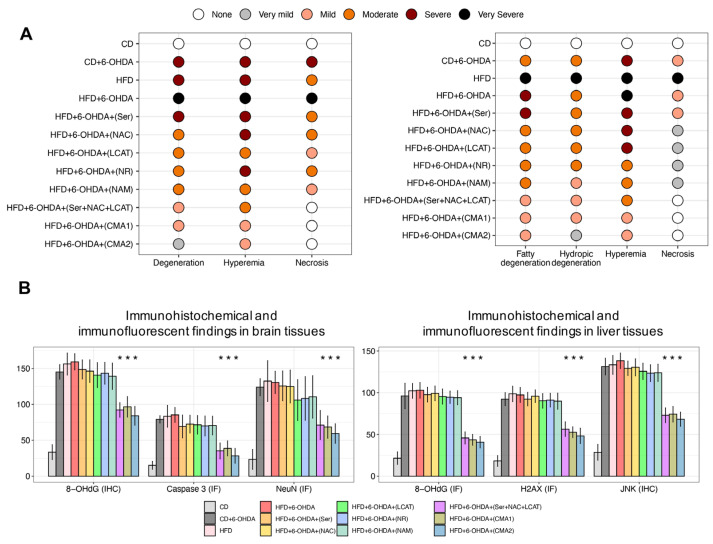
Immunohistopathological examination of brain and liver tissues in PD-like animal model. (**A**) Histopathological image analysis results of rat brain and liver tissue. Slides evaluated by two independent pathologists and immunopositivity scores were: None (−), very mild (+), mild (++), moderate (+++), severe (++++), and very severe (+++++). (**B**) Bar plot shows immunohistochemical and immunofluorescent findings in brain and liver tissues. Asterisk (***) denotes statistical significance (*p* < 0.05). The difference and *p*-value are estimated by one-way ANOVA or Mann–Whitney U test. CD: chow diet, 6-OHDA: 6-hydroxydopamine hydrochloride, HFD: high-fat diet, Ser: L-serine, NAC: N-acetyl-L-cysteine, LCAT: L-carnitine tartrate, NR: nicotinamide riboside, NAM: nicotinamide, CMA1: Combined Metabolic Activators 1, CMA2: Combined Metabolic Activators 2, IF: immunofluorescence; IHC: immunohistochemistry. The results from CMA1 and its individual components have been reported earlier [35].

In an analysis of brain tissue samples, it was observed that subjects on HFD who also received 6-OHDA exhibited extensive neuronal degradation, blood vessel congestion, and cell death. Conversely, subjects treated with either CMA1 and CMA or its individual constituents experienced only slight neuronal degradation and vascular congestion, with no evidence of cell death observed in brain neurons (Figure 4A and Figure S2, Dataset S2). Further, liver tissue examinations were conducted based on the premise that CMA2 might enhance metabolic parameters, potentially leading to better neurological function. In subjects fed a HFD and given 6-OHDA, there was notable fatty and watery degeneration of liver cells, severe vascular congestion, and some cell death in liver vessels. Post-treatment with CMA or its separate components, there was a notable reduction in the severity of both types of liver cell degeneration and vascular congestion, with no cell death in liver vessels (Figure 4A and Figure S2, Dataset S2). These changes were statistically significant (*p* < 0.05) when compared to the HFD subjects treated with 6-OHDA.

Immunohistochemical analysis of brain specimens from animals on a HFD and subjected to 6-OHDA revealed significantly higher levels of 8-OHdG, NeuN, and Caspase 3 compared to those on a CD (Figure 4B and Figure S2, Dataset S2). Analysis of brain tissues from animals treated with either individual metabolic activators or CMA exhibited only minimal levels of 8-OHdG and localized NeuN, caspase 3 expression, particularly in the group treated with CMA2 (Figure 4B and Figure S2). There was a notable statistical difference (*p* < 0.05) between the metabolic activator or CMA-treated groups and the HFD/6-OHDA group. In a similar vein, liver specimens from the HFD/6-OHDA animals displayed a pronounced increase in JNK, 8-OHdG, and H2AX expression when contrasted with CD-fed control animals (Figure 4B and Figure S2, Dataset S2). Liver tissues from those treated with metabolic activators or CMA showed considerably lower expression levels of JNK, 8-OHdG, and H2AX (Figure 4B and Figure S2, Dataset S2). There was a significant statistical difference (*p* < 0.05) when comparing the effects of treatments with either metabolic activators or CMA against those of the HFD + 6-OHDA group. Overall, our studies suggest that both CMAs can enhance neurologic and metabolic health in animal models, with CMA2 showing superior benefits in both brain and liver tissues.

### 3.3. Both CMA1 and CMA2 Shows a Beneficial Effect on Behavioural Functions in PD Rats

Animals on HFD demonstrated contralateral rotation, a response not seen in those on CD. Following unilateral lesions induced by 6-OHDA, subjects showed rotational behavior when treated with dopaminergic drugs. On average, the HFD + 6-OHDA group exhibited 303.33 contralateral rotations, compared to 183.33 rotations in the group treated with 6-OHDA alone. The introduction of separate metabolic activators reduced this contralateral rotation at different rates. All treatment groups showed statistically significant decreases when compared to the HFD + 6-OHDA group (*p* < 0.001, Dataset S2).

**Figure 5:**
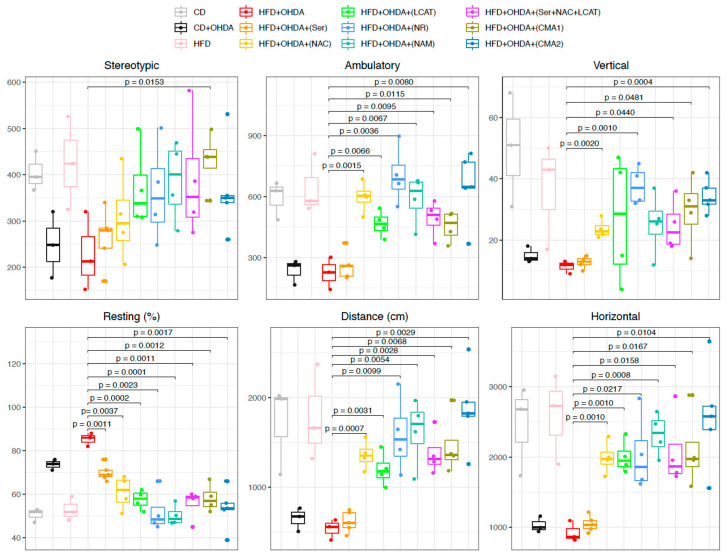
Administration of CMA shows beneficial effects on behavioral functions in PD-like animal models. Boxplot showing the changes in locomotor activity in each group. The comparison was applied between the treated groups and the HFD plus 6-OHDA group. CD: chow diet, 6-OHDA: 6-hydroxydopamine hydrochloride, HFD: high-fat diet, Ser: L-serine, NAC: N-acetyl-L-cysteine, LCAT: L-carnitine tartrate, NR: nicotinamide riboside, NAM: nicotinamide, CMA1: Combined Metabolic Activators 1, CMA2: Combined Metabolic Activators 2. The results from CMA1 and its individual components have been reported earlier [35].

The analysis of animal motor functions was conducted through a locomotor activity examination. Our data indicated that metabolic activators markedly improved stereotypy in comparison to control groups (Table 1 and Figure 5 and Figure S3), with the hierarchy of treatment effectiveness for reducing stereotypy being: CMA1 > L-serine + NAC + L-CAT > L-CAT > CMA2 > NR > NAC > L-serine. Similarly, supplementation with various metabolic cofactors enhanced ambulatory activity (Table 1 and Figure 5 and Figure S3). Rats subjected to 6-OHDA and HFD, or 6-OHDA alone, showed a significant (*p* < 0.05) increase in resting time compared to the control group. Conversely, administration of either individual metabolic cofactors or CMA reduced the resting time (Table 1 and Figure 5 and Figure S3). Moreover, the administration of metabolic activators, either in combination or individually (except L-serine), led to improvements in traveled distances and horizontal movement. Notably, rodents treated with CMA2 exhibited the most significant improvement in both traveled distance and horizontal activity (Table 1 and Figure 5 and Figure S3).

## 4. Discussion

While neurodegenerative diseases can manifest in a variety of ways, they generally share certain features, such as a gradual worsening of symptoms and similar underlying molecular pathologies. Animal models play a crucial role in understanding these complex diseases due to the challenge of replicating the complexities of the human nervous system in experimental settings. In our study, we assessed the impact of CMAs on animals modeled to mimic AD and PD. Our findings indicate that both CMA1 and CMA2 not only improve metabolic conditions and behavioral performance but also correlate with positive neuro-histological changes. Moreover, we found that CMA2 showed a superior effect compared to CMA1. 

NAD+, a crucial molecule in the body, is essential for maintaining cellular energy balance, responding to stress, and ensuring cell survival [38]. It influences neural adaptability and stress resistance through a variety of NAD+-dependent enzymes [39]. Enhancement of NAD+ synthesis through nicotinamide supplementation has been shown to bolster cellular energy metabolism and facilitate neuroprotection. This, in turn, mitigates oxidative stress, a significant factor in the pathophysiology of NDDs. Moreover, nicotinamide supports DNA repair mechanisms and promotes synaptic plasticity, enhancing neuronal resilience and cognitive performance [40]. Studies on animal models of NDDs have shown that nicotinamide supplementation has demonstrated efficacy in preventing DNA fragmentation in neurons subjected to oxidative stress, thereby safeguarding against apoptosis or necrosis [41]. This protective mechanism is attributed to the elevation of NAD+ levels in the brain by up to 50%, offering a buffer against NAD+ depletion and supporting DNA repair processes [41]. Enhancing NAD+ levels appears to counteract the decline in brain energy metabolism and oxidative damage associated with cognitive deterioration. Studies suggest that nicotinamide, by maintaining intracellular NAD+ levels, can mitigate age-related cellular damage and neurodegeneration, offering a promising therapeutic avenue. For instance, nicotinamide has been shown to restore cognitive deficits in Alzheimer’s disease models by modulating tau phosphorylation and enhancing mitochondrial function and autophagy, processes vital for neuronal health [42,43]. It also appears to protect against amyloid β-induced oxidative damage and mitochondrial dysfunction in synaptic structures, critical for memory and learning [44]. Additionally, addressing compartmentalized redox imbalances through dietary nicotinamide supplementation could counteract aging-related cognitive decline and neurodegenerative diseases by optimizing energy and methyl group availability, which are essential for brain development and function [45]. Collectively, these findings underscore the potential of nicotinamide in preventing or delaying the onset of neurodegenerative diseases by targeting multiple pathological pathways, from enhancing mitochondrial health and stimulating mitophagy to reducing oxidative stress and improving bioenergetic deficits.

An imbalance between the production of reactive oxygen species within cells and the antioxidant defense mechanisms can lead to oxidative stress, which in turn may cause memory deficits. The reduction in synaptic reorganization within neural networks is known to contribute to cognitive issues as people age, but it is not yet clear if oxidative stress regulates this process or which specific pathways are involved. Maintaining redox balance in older animals seems to offer protection against cognitive deterioration. Long-term administration of NAC has been observed to mitigate oxidative damage in the hippocampus of elderly rats [5]. Likewise, treatment with NAC has also demonstrated a protective effect on dopaminergic neuron decline and the neuroinflammatory response in the nigrostriatal pathway in an animal study [46]. As neurodegenerative diseases progress, developing neuroprotective methods is increasingly essential for decelerating damage to dopaminergic cells and related inflammation. Our research lays the foundation for further studies to verify the potential effectiveness of such neuroprotective strategies.

The therapeutic potential of L-carnitine in enhancing mitochondrial health and protecting against neurodegenerative pathologies offers new avenues for the management of these debilitating diseases. In the context of PD, comprehensive analyses have revealed significant decreases in long-chain acylcarnitines, suggesting an initial suppression of mitochondrial β-oxidation and identifying these metabolites as promising diagnostic biomarkers [47]. Similarly, mitochondrial dysfunction, characterized by the inability to maintain metabolic flexibility and energy production, is a common pathway in neurodegenerative diseases, with L-carnitine playing a pivotal role in supporting mitochondrial function through fatty acid oxidation and in mitigating oxidative stress and neurotoxicity [48]. This is further supported by studies on AD, where amyloid beta-peptides cause neuronal injury through excitotoxic mechanisms, with L-carnitine and its derivatives offering neuroprotective effects by enhancing mitochondrial function, reducing oxidative stress, and improving neuronal viability [49,50,51]. 

An imbalance in brain aerobic glycolysis is often observed in the early phases of AD. The connection between this metabolic disturbance and the resulting structural and cognitive deficits remains unclear. There is increasing evidence that L-serine plays a role in the brain’s release of various cytokines, which helps to restore cognitive abilities and provide neuroprotection against neurological damage [52,53,54,55,56]. One recent study has suggested that astrocytic glycolysis might influence cognitive functions and points to oral L-serine as a potential treatment option for AD [10]. Our research has concluded that L-serine supplementation can significantly enhance both metabolic functions and histopathological conditions in animal models that resemble AD and PD.

Here, we have demonstrated that a compound called CMA2 enhances mitochondrial function, leading to reduced inflammation, cellular degradation, and death in neuronal cells. This has shown promising results in improving the behaviors of mouse models of NDDs, indicating its potential as a therapeutic candidate suitable for advancing to human trials. Furthermore, our findings lay the groundwork for future research into targeting specific metabolic pathways to increase NAD+ and glutathione levels. These avenues may pave the way for new treatments aimed at adjusting mitochondrial activity in NDDs by encouraging the metabolism of fatty acids within the mitochondria, a key factor for an effective mitochondrial response to the challenges posed by neurodegenerative conditions.

In conclusion, we showed through relevant metabolic models for AD and PD that administering both CMA1 and CMA2 leads to improved behavioral results, which correspond with enhanced neuro-histological findings in both the brain and liver. CMA2 also showed a superior effect compared to CMA1 and other individual metabolic activators. The considerable positive impacts on both histological and clinical conditions following CMA2 therapy underscore the critical role of modulating multiple metabolic routes in neuroprotective treatment strategies for NDDs.

## Figures and Tables

**Table 1:** Comprehensive behavioral analysis of rats treated with HFD, 6-OHDA and different metabolic activators in the animal model of PD. The results from CMA1 and its individual components have been reported earlier [35].

Groups	Stereotypic Movement	Ambulatory Movement	Vertical Activity	Horizontal Activity	Distance Traveled (cm)	Resting (%)
CD	404.25 ± 42.70	593.75 ± 95.05	50.00 ± 18.50	24.50 ± 6.40	1715.50 ± 495.90	50.75 ± 3.20
HFD	425.00 ± 100.50	643.75 ± 146.05	36.75 ± 17.40	26.00 ± 6.30	1784.00 ± 537.20	53.00 ± 5.60
CD + 6-OHDA	248.25 ± 71.50	236.00 ± 61.30	15.00 ± 2.60	10.25 ± 1.30	650.00 ± 131.30	73.75 ± 2.50
HFD + 6-OHDA	228.25 ± 85.00	224.75 ± 79.05	11.25 ± 2.10	9.75 ± 1.50	536.00 ± 113.90	85.25 ± 3.10
HFD + 6-OHDA + Ser	263.00 ± 62.70	260.75 ± 68.25 *	12.75 ± 1.90	10.50 ± 1.20	616.75 ± 118.80	70.00 ± 3.80 *
HFD + 6-OHDA +NAC	307.75 ± 96.05	598.00 ± 76.3 *	23.75 ± 3.10 *	19.75 ± 2.30 *	1359.00 ± 159.80 *	51.25 ± 4.70 *
HFD + 6-OHDA + LCAT	370.75 ± 89.60	466.00 ± 64.4 5*	27.00 ± 4.80	20.00 ± 2.30 *	1199.25 ± 188.50 *	57.50 ± 4.40 *
HFD + 6-OHDA +NR	361.75 ± 108.10	704.75 ± 144.10 *	37.75 ± 6.30 *	20.50 ± 5.50 *	1587.50 ± 429.20 *	49.50 ± 11.40 *
HFD + 6-OHDA +NAM	387.25 ± 87.05	587.00 ± 121.70 *	25.25 ± 10.20 *	23.25 ± 3.10 *	1619.00 ± 379.10 *	50.25 ± 4.70 *
HFD + 6-OHDA+Ser + NAC + LCAT	390.50 ± 135.50	492.25 ± 90.05 *	24.75 ± 8.30 *	20.75 ± 5.30 *	1380.25 ± 243.80 *	55.50 ± 7.10 *
HFD + 6-OHDA +CMA1	429.75 ± 63.70 *	453.75 ± 75.80 *	29.50 ± 11.60 *	21.00 ± 5.40 *	1470.00 ± 343.80 *	58.25 ± 6.50 *
HFD + 6-OHDA +CMA2	367.20 ± 99.40 *	648.00 ± 173.40 *	34.50 ± 5.30 *	25.75 ± 7.40 *	1873.00 ± 456.30 *	53.50 ± 9.70 *

The difference between individual and combined metabolic activators-treated group and HFD plus 6-OHDA group. Statistical significance is derived from one-way ANOVA or Mann–Whitney U test and Asterisk (*) denotes *p* < 0.05. CD: chow diet, 6-OHDA: 6-hydroxydopamine hydrochloride, HFD: high-fat diet, Ser: L-serine, NAC: N-acetyl-L-cysteine, LCAT: L-carnitine tartrate, NR: nicotinamide riboside, NAM: nicotinamide, CMA1: Combined Metabolic Activators 1, CMA2: Combined Metabolic Activators 2.

## Data Availability

All codes used for the analyses are available at https://github.com/sysmedicine/NDDanimal.

## Supplementary Materials

The following supporting information can be downloaded at: https://www.mdpi.com/article/10.3390/biomedicines12040927/s1, Dataset S1: Characteristics of animals and statistical analysis results of AD animal study; Dataset S2: Characteristics of animals and statistical analysis results of PD animal study. Figure S1: Histopathological, immunohistochemical and immunofluorescence images of rat tissues in the AD-like animal model; Figure S2: Histopathological, immunohistochemical and immunofluorescence images of rat tissues in the PD-like animal model; Figure S3: Activity meter graph shows behavioral functions in PD-like animal model.
