# Supplementary material for: Combined Metabolic Activators with Different NAD+ Precursors Improve Metabolic Functions in the Animal Models of Neurodegenerative Diseases
Source: Biomedicines. 2024 Apr 22;12(4):927. doi: 10.3390/biomedicines12040927 (PMC11048203; doi:10.3390/biomedicines12040927)
Supplement: Supplementary file 1 [file biomedicines-12-00927-s001.zip › Figure Legends.pdf]

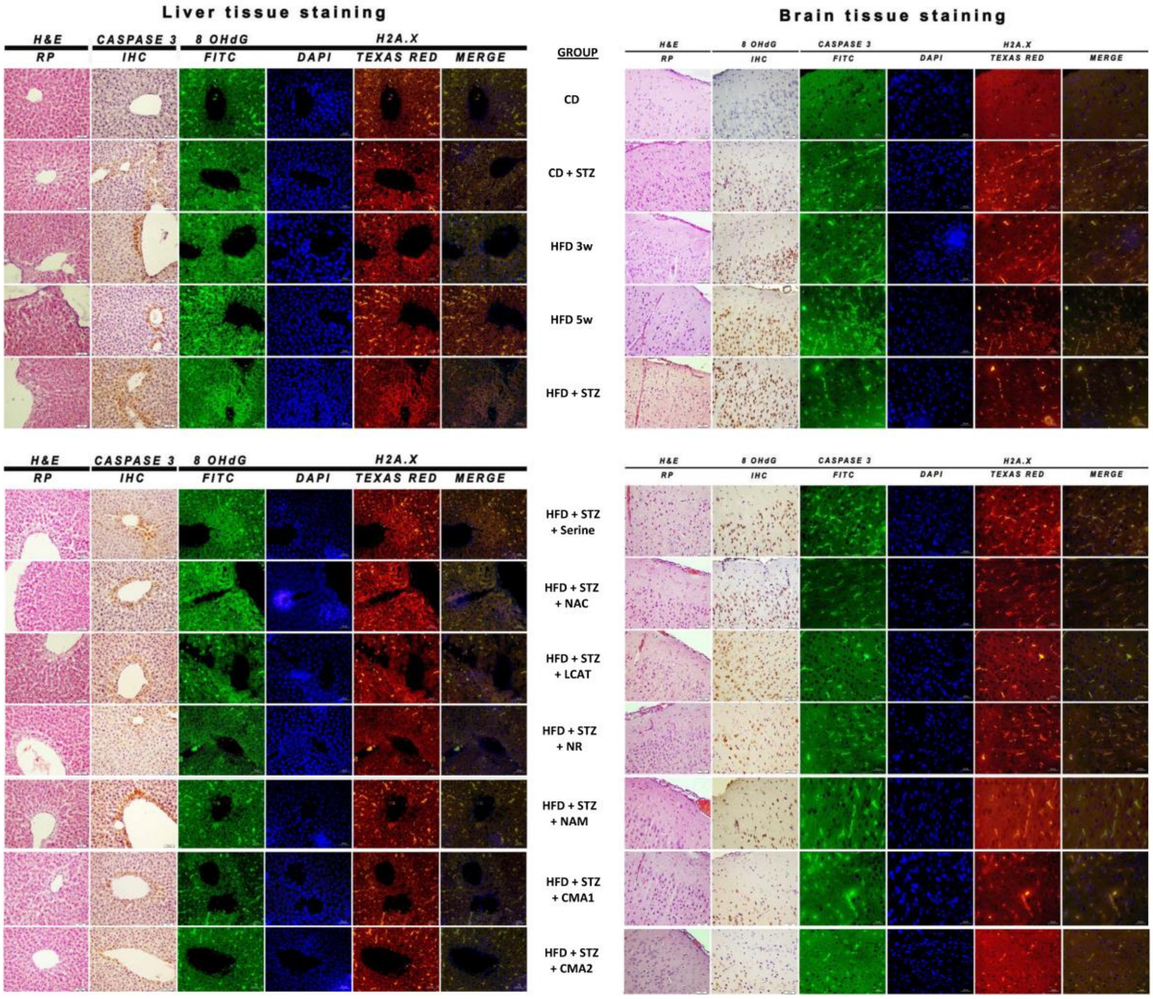

Figure S1 legend:

Histopathological (Haematoxylin &Eosin), immunohistochemical (Caspase 3 for liver, 8-OHdG for brain) and immunofluorescence (FITC, DAPI, Texas Red and merged) images of rat brain (antibodies: Caspase 3 and H2A.X) and liver (antibodies: 8-OHdG and H2A.X) tissues. Brain tissue (cerebral cortex) is presented at right side and liver tissue (acinar regions) at left side. Slides evaluated by two independent pathologists and histopathological scores were: None (-), very mild (+), mild (++), moderate (+++) and severe (++++)

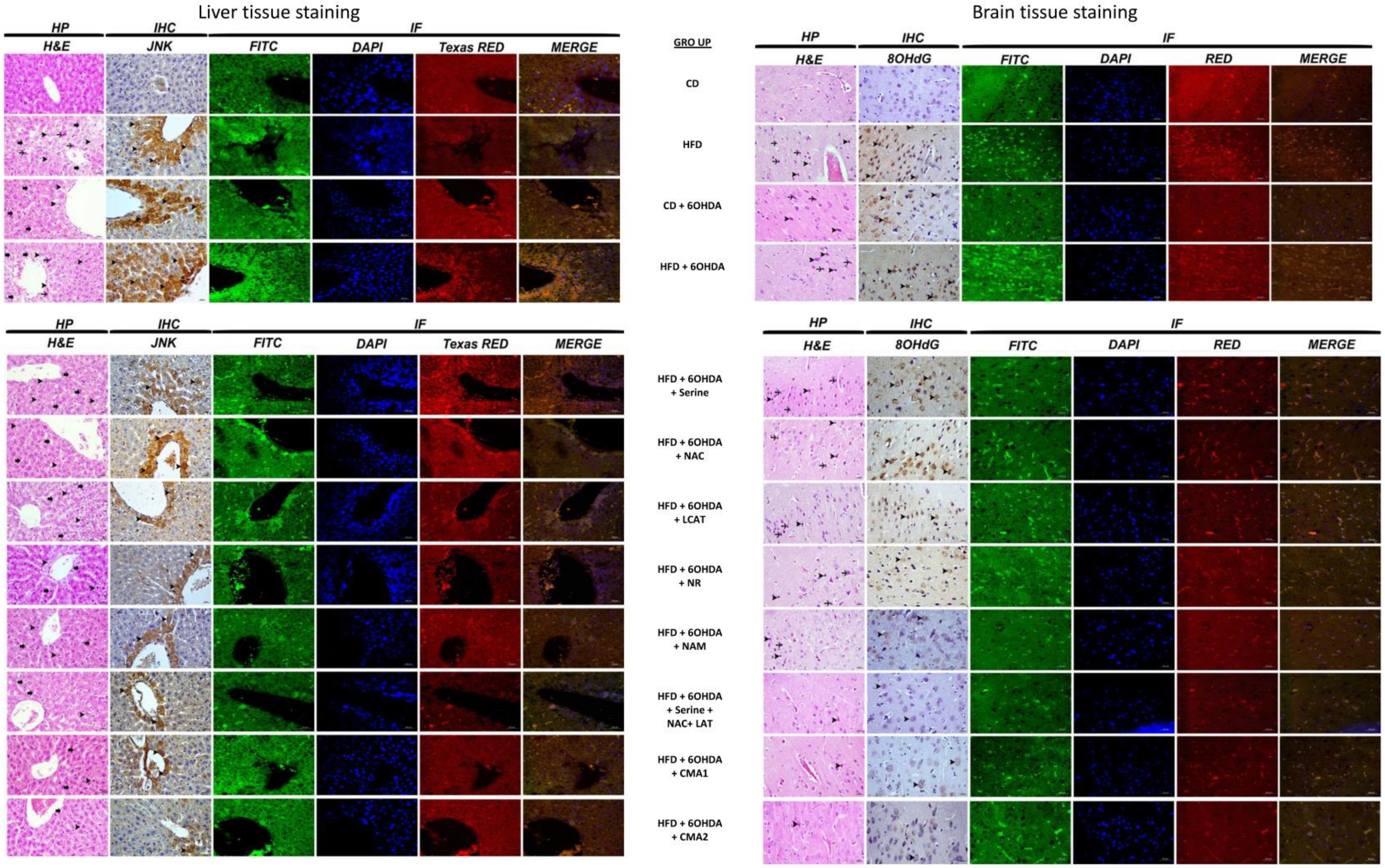

Figure S2 legend:

Histopathological (Haematoxylin &Eosin), immunohistochemical (JNK for liver, 8-OHdG for brain) and immunofluorescence (FITC, DAPI, Texas Red and merged) images of rat brain (antibodies: Caspase 3 and NeuN) and liver (antibodies: 8-OHdG and H2A.X) tissues. Brain tissue (cerebral cortex) is presented at right side and liver tissue (acinar regions) at left side. Slides evaluated by two independent pathologists and histopathological scores were: None (-), very mild (+), mild (++), moderate (+++) and severe (++++)

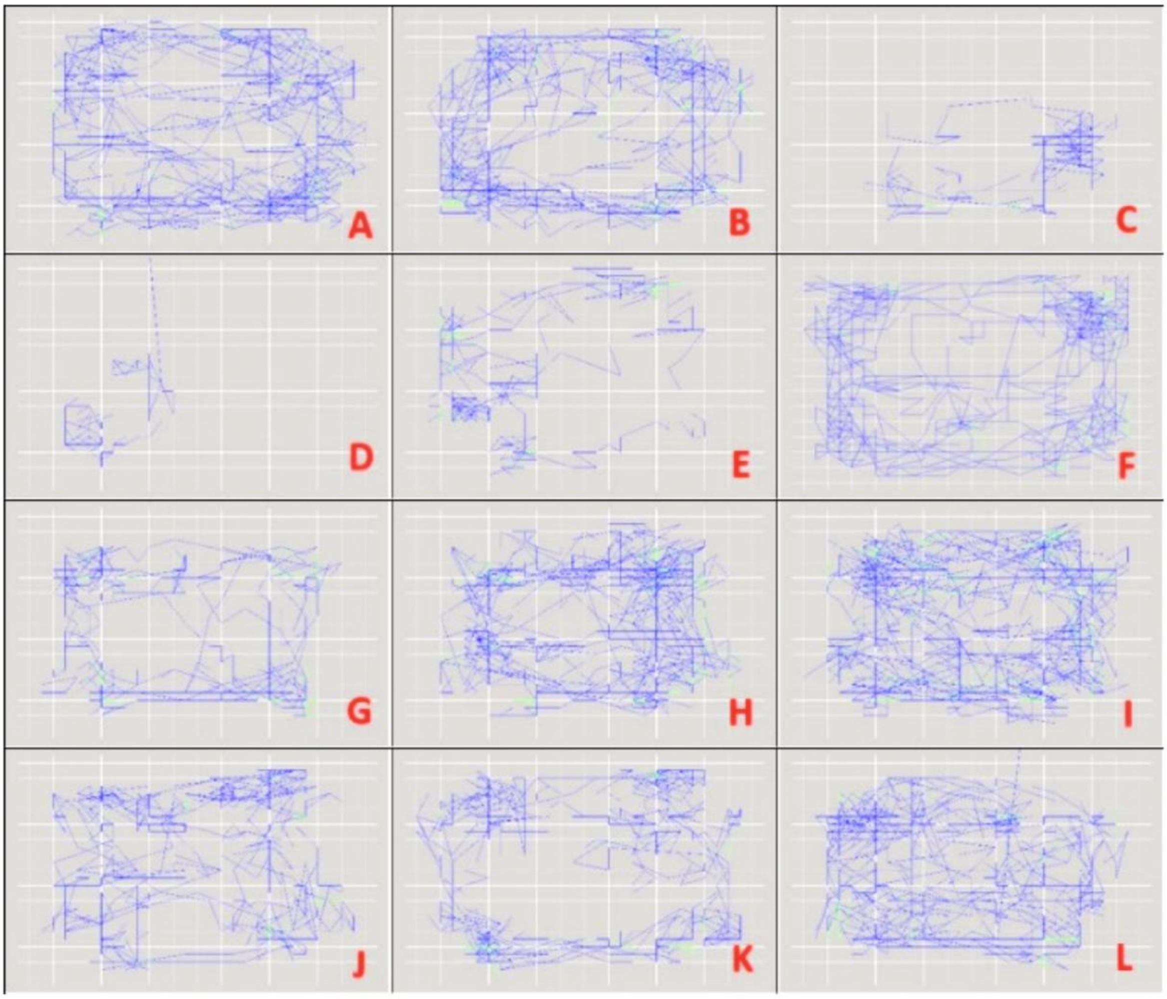

Figure S3 Legend:

The activitymeter graph consists of an image to represent the total locomotor activity of each treatment type. Different letters above the columns present significant differences ($p < 0.05$). The comparison was applied between treated groups and HFD plus 6-OHDA group. A: CD, B: HFD, C: 6-OHDA, D: HFD + 6-OHDA, E: Ser, F: NAC, G: L-CAT, H: NR, I: NAM, J: Ser + NAC + L-CAT, K: Ser + NAC+ L-CAT + NR, L: Ser + NAC+ L-CAT + NAM. CD: chow diet, 6-OHDA: 6-hydroxydopamine hydrochloride, HFD: high-fat diet, Ser: L-serine, NAC: N-acetyl-L-cysteine, LCAT: L-carnitine tartrate, NR: nicotinamide riboside, NAM: nicotinamide.
